# SO_2_ Poisoning of Cu-CHA deNO_*x*_ Catalyst: The Most Vulnerable Cu Species Identified by X-ray Absorption Spectroscopy

**DOI:** 10.1021/jacsau.2c00053

**Published:** 2022-04-11

**Authors:** Anastasia Yu. Molokova, Elisa Borfecchia, Andrea Martini, Ilia A. Pankin, Cesare Atzori, Olivier Mathon, Silvia Bordiga, Fei Wen, Peter N. R. Vennestrøm, Gloria Berlier, Ton V. W. Janssens, Kirill A. Lomachenko

**Affiliations:** † European Synchrotron Radiation Facility, 71 avenue des Martyrs, CS 40220, 38043 Grenoble Cedex 9, France; ‡ Department of Chemistry and NIS Centre, University of Turin, via Giuria 7,10125 Turin, Italy; § The Smart Materials Research Institute, Southern Federal University, Sladkova 174/28, 344090 Rostov-on-Don, Russia; ∥ Umicore AG & Co, Rodenbacher Chaussee 4, 63457 Hanau, Germany; ⊥ Umicore Denmark ApS, Kogle Allé 1, 2970 Hørsholm, Denmark

**Keywords:** selective catalytic reduction, Cu-CHA, deNO_*x*_ catalysis, sulfur poisoning, X-ray absorption spectroscopy, X-ray adsorbate quantification, XAS, XAQ

## Abstract

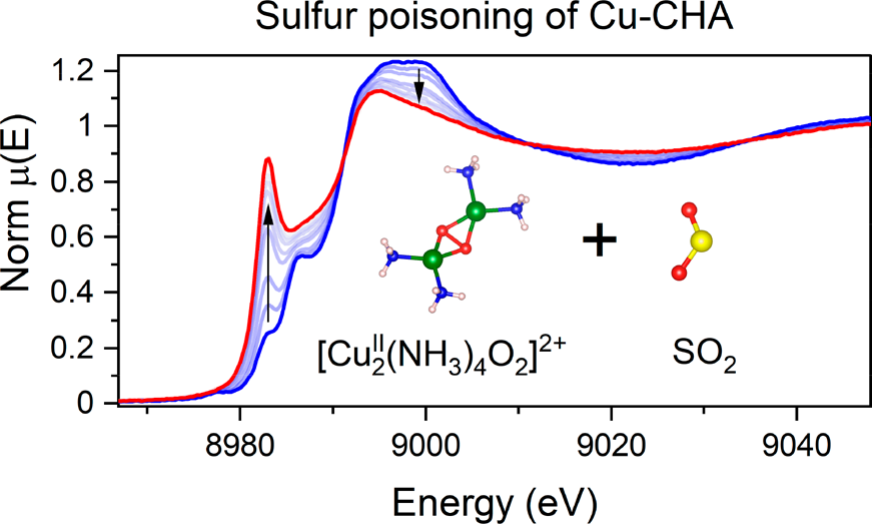

Cu-exchanged chabazite zeolites (Cu-CHA) are effective catalysts for the NH_3_-assisted selective catalytic reduction of NO (NH_3_-SCR) for the abatement of NO_*x*_ emission from diesel vehicles. However, the presence of a small amount of SO_2_ in diesel exhaust gases leads to a severe reduction in the low-temperature activity of these catalysts. To shed light on the nature of such deactivation, we characterized a Cu-CHA catalyst under well-defined exposures to SO_2_ using *in situ* X-ray absorption spectroscopy. By varying the pretreatment procedure prior to the SO_2_ exposure, we have selectively prepared Cu^I^ and Cu^II^ species with different ligations, which are relevant for the NH_3_-SCR reaction. The highest reactivity toward SO_2_ was observed for Cu^II^ species coordinated to both NH_3_ and extraframework oxygen, in particular for [Cu^II^_2_(NH_3_)_4_O_2_]^2+^ complexes. Cu species without either ammonia or extraframework oxygen ligands were much less reactive, and the associated SO_2_ uptake was significantly lower. These results explain why SO_2_ mostly affects the low-temperature activity of Cu-CHA catalysts, since the dimeric complex [Cu^II^_2_(NH_3_)_4_O_2_]^2+^ is a crucial intermediate in the low-temperature NH_3_-SCR catalytic cycle.

The emission of nitrogen oxides (NO_*x*_) from diesel vehicles is a global environmental challenge.^1,2^ State of the art exhaust gas aftertreatment systems contain catalysts for selective catalytic reduction of NO_*x*_ by ammonia (NH_3_-SCR), capable of reducing well over 90% of the NO_*x*_ emitted by the engine. In the NH_3_-SCR reaction, NO reacts with NH_3_ in the presence of O_2_ to form N_2_ and H_2_O. At present, Cu-exchanged chabazites (Cu-CHA) are the preferred catalysts for NH_3_-SCR, due to their superior low-temperature activity (150–350 °C)^3,4^ and hydrothermal stability.^5,6^ The temperature dependence of the NH_3_-SCR activity of Cu-CHA catalysts shows a minimum at around 350 °C, which indicates that the reaction mechanism at low temperatures is different from that at higher temperatures.^7^

The NH_3_-SCR reaction cycle for the low-temperature activity is a redox cycle, consisting of a series of oxidation and reduction steps, in which the oxidation state of Cu changes between Cu^I^ and Cu^II^. The NO and NH_3_ coordinate to Cu in the zeolite, giving rise to a variety of Cu species along the NH_3_-SCR cycle.^8−11^ The low-temperature activity of Cu-CHA catalysts originates from the ability to form mobile Cu^I^(NH_3_)_2_ complexes under SCR conditions. Pairs of these species constitute the active Cu sites capable of O_2_ activation via the formation of [Cu^II^_2_(NH_3_)_4_O_2_]^2+^ dimers around 200 °C, which is a crucial step in the NH_3_-SCR reaction cycle.^12,13^

In practice, the application of Cu-CHA catalysts for the NH_3_-SCR is restricted to ultralow-sulfur diesel fuels, due to the fact that a few ppm of SO_2_ present in the exhaust gas drastically reduces the activity at low temperatures.^3,4,14^ Multiple studies show that SO_2_ affects the Cu mobility, the amount of Cu active sites,^14^ and the redox behavior of the Cu in the NH_3_-SCR cycle.^9,15^ Most studies have focused on the overall effect of SO_2_ on the performance of the catalysts,^14−21^ while the chemistry behind SO_2_ poisoning at the molecular level remains poorly understood. To determine a mechanism for SO_2_ poisoning, one must identify the species in the Cu-CHA catalysts that interact with SO_2_. To this end, we have selectively prepared well-defined Cu^I^ and Cu^II^ species with different ligands inside the pores of the Cu-CHA catalyst and exposed them to SO_2_ under well-defined conditions. We monitored the changes in the Cu K-edge X-ray absorption spectra (XAS) during the absorption of SO_2_. This allowed us to determine the chemical state of the Cu that interacts with SO_2_. The results were corroborated by X-ray emission spectroscopy (XES) and measurements of the SO_2_ uptake using temperature-programmed desorption (TPD) of SO_2_.

The Cu-CHA catalyst used in this study had a Si/Al ratio of 6.7 and a Cu loading of 3.2 wt % (Cu/Al = 0.24). The Cu K-edge XAS and Cu Kβ valence-to-core XES measurements were carried out at the BM23^22^ and ID26^23^ beamlines of the European Synchrotron Radiation Facility (ESRF), respectively. Sample treatment protocols consisted of three distinct steps. First, all samples were heated to 550 °C in a 10% O_2_/He flow, removing water and forming Cu^II^ species bound to the framework of the zeolite (fw-Cu^II^). Then, the specific state of Cu was prepared, using one of the six different pretreatment procedures summarized in Table 1. Finally, the catalyst was exposed to 400 ppm SO_2_/He flow at 200 °C for 3 h until no visible changes in the spectra occurred. Further experimental details are given in the Supporting Information.

**Table 1:** Pretreatment Procedures and Resulting Cu Species

procedure	conditions	dominant Cu state	designation in the text and figures	ref
1	1% H_2_ at 400 °C; cooling to 200 °C in He	fw-Cu^I^	fw-Cu^I^	(24)
2	500 ppm of NO + 600 ppm of NH_3_ at 200 °C	mobile [Cu^I^(NH_3_)_2_]^+^	[Cu^I^(NH_3_)_2_]^+^	(24)
3	500 ppm of NO + 600 ppm of NH_3_ at 200 °C; heating to 550 °C in He; cooling back to 200 °C in He	fw-Cu^I^ (after thermal treatment of [Cu^I^(NH_3_)_2_]^+^)	[Cu^I^(NH_3_)_2_]^+^ + T	(24)
4	10% O_2_ at 200 °C	fw-Cu^II^	fw-Cu^II^	(25)
5	500 ppm of NO + 600 ppm of NH_3_ at 200 °C; He purge; 10% O_2_ at 200 °C	mobile [Cu^II^_2_(NH_3_)_4_O_2_]^2+^ dimer	[Cu^II^_2_(NH_3_)_4_O_2_]^2+^	(13)
6	600 ppm of NH_3_ at 200 °C	mixed^a^	Cu^II^ + NH_3_	this work

a Procedure 6 results in a mixture of two NH_3_-coordinated Cu species, as discussed further in the text.

The Cu species formed with the pretreatments differ in three aspects: (1) the oxidation state of Cu (Cu^I^ or Cu^II^), (2) the coordination of the Cu (NH_3_ or/and O), and (3) the interaction of the Cu with the framework (fw-coordinated or mobile species).

Figure 1 shows the evolution of Cu K-edge XANES and EXAFS spectra during the exposure of the pretreated Cu-CHA catalyst to 400 ppm SO_2_/He flow at 200 °C. For all Cu^I^ species and fw-Cu^II^ species (procedures 1–4 in Table 1), only minor changes are observed upon SO_2_ exposure, indicating that these species are not very reactive toward SO_2_. In contrast, for Cu^II^ species in the presence of NH_3_ (procedures 5 and 6) significant changes are observed in the spectra. In these cases, the exposure to SO_2_ results in a pronounced increase of the XANES peak at 8983 eV, characteristic for linear Cu^I^ complexes,^9,26,27^ and a decrease in the intensity of the first shell in the EXAFS FT. This means that some of the Cu^II^ species are reduced to Cu^I^ upon interaction with SO_2_. The decrease in the first-shell intensity indicates a reduction of the coordination number for the Cu ions, which is also in line with the formation of a linear Cu^I^ species.

**Figure 1:**
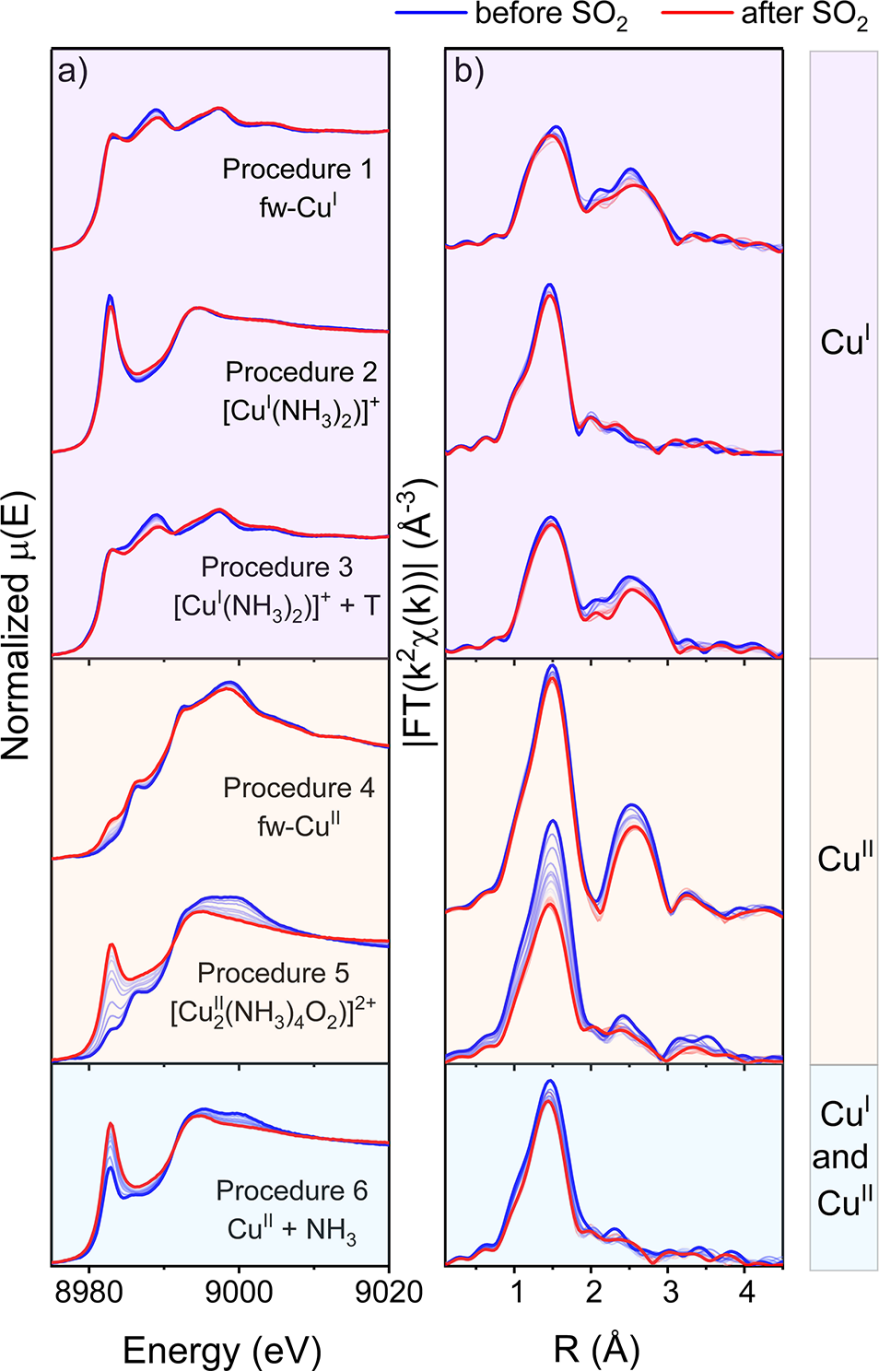
Cu K-edge XANES (a) and FT-EXAFS spectra (b) collected *in situ* during the exposure of Cu species obtained in procedures 1–6 to SO_2_ at 200 °C.

The species obtained in procedure 5 are the oxygen-bridged diamine dicopper complexes [Cu^II^_2_(NH_3_)_4_O_2_]^2+^, which are formed by the reaction of O_2_ with a pair of [Cu^I^(NH_3_)_2_]^+^ complexes.^13^ In the reaction cycle for the low-temperature NH_3_-SCR reaction,^11,13,28^ the [Cu^II^_2_(NH_3_)_4_O_2_]^2+^ complexes react with NO, which eventually leads to the production of N_2_ and H_2_O. The observation that the [Cu^II^_2_(NH_3_)_4_O_2_]^2+^ complexes are reactive toward SO_2_ is therefore a good explanation for the SO_2_-induced deactivation of Cu-CHA catalysts for NH_3_-SCR: the reaction with SO_2_ interrupts the NH_3_-SCR cycle, thereby decreasing the activity of the catalyst.

The other case where Cu reacts with SO_2_ is obtained in procedure 6 by exposure of the fw-Cu^II^ species to NH_3_. Previously, a similar pretreatment resulted in a mixture of linear [Cu^I^(NH_3_)_2_]^+^ and either square-planar [Cu^II^(NH_3_)_4_]^2+^ complexes or mixed-ligand [Cu^II^O_*x*_(NH_3_)_*y*_]^2+^ moieties.^9,24^ For the sample reported in this work, the mixed-ligand configuration is more likely. Indeed, linear combination fits of the XANES data on the basis of references for the linear [Cu^I^(NH_3_)_2_]^+^ complex and pure Cu^II^(NH_3_)_4_ groups (aqueous [Cu^II^(NH_3_)_4_]^2+^ or solid-state [Cu^II^(NH_3_)_4_]SO_4_·H_2_O) resulted in visible discrepancies with the data (Figure S7 in the Supporting Information). A better agreement is obtained when the spectrum of oxygen-bridged diamine dicopper complex [Cu^II^_2_(NH_3_)_4_O_2_]^2+^ is used as a Cu^II^ reference in combination with [Cu^I^(NH_3_)_2_]^+^, with approximately equal weights for each component (Figure 2). The necessary stock of available oxygen needed for the formation of the mixed-ligand species is expected to be present in the sample, as a wavelet analysis of the EXAFS collected after heating to 550 °C and cooling to 200 °C in 10% O_2_/He flow reveals the presence of Cu–Cu scattering usually attributed to the oxygen-containing dimers^29,30^ (Figure S8 in the Supporting Information), which may be susceptible to form mixed-ligand species upon exposure to NH_3_.

**Figure 2:**
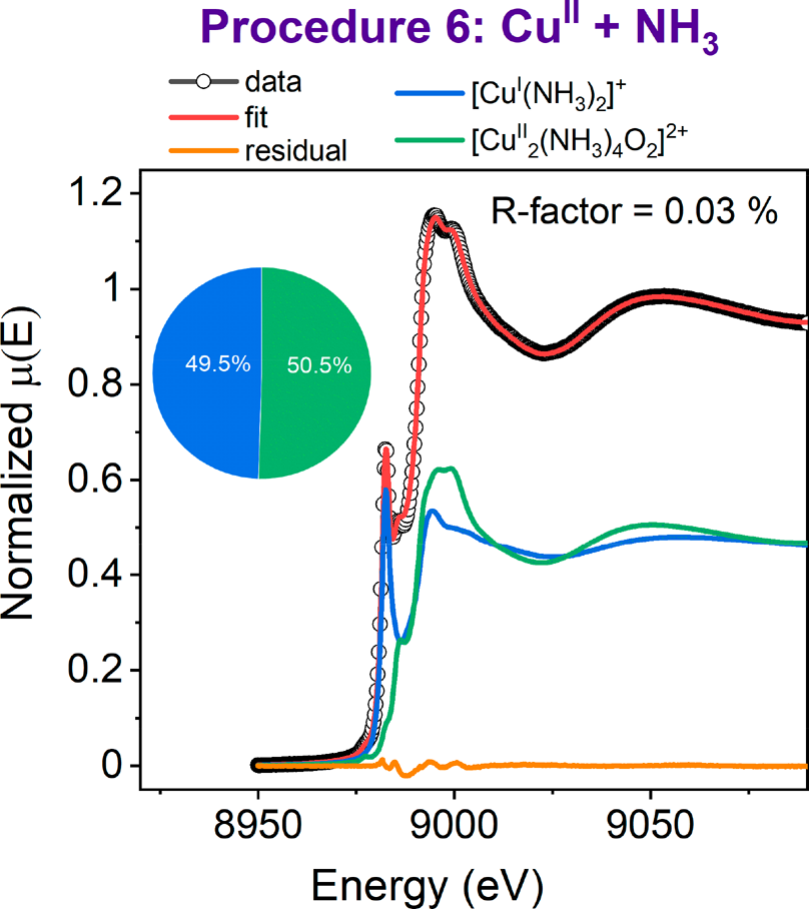
Linear combination fit of Cu K-edge XANES spectra obtained in Cu-CHA after exposing fw-Cu^II^ species to NH_3_ at 200 °C (Cu^II^ + NH_3_ pretreatment).

The evolution of XANES spectra upon interaction with SO_2_ shows that the most susceptible species are Cu^II^ with mixed (NH_3_)_*x*_O_*y*_ ligation, whereas Cu^I^ species or Cu^II^ in the absence of NH_3_ are much less affected. These findings are supported by X-ray adsorbate quantification (XAQ) data,^31^ collected simultaneously with the XAS measurements during the exposure to SO_2_, and a TPD analysis of a parallel set of catalyst samples, exposed to the same pretreatments used in XANES experiments (Figure 3a). We find the highest sulfur content (S/Cu ratio) for the [Cu^II^_2_(NH_3_)_4_O_2_]^2+^ and Cu^II^ + NH_3_ procedures. The sulfur uptake of the [Cu^I^(NH_3_)_2_]^+^ and fw-Cu^II^ moieties was ca. 3 times lower, and for the bare fw-Cu^I^ species, it was ca. 6 times lower. These results show that the reaction between the [Cu^II^_2_(NH_3_)_4_O_2_]^2+^ species and SO_2_ contributes the most to the accumulation of SO_2_ in the Cu-CHA catalyst.

**Figure 3:**
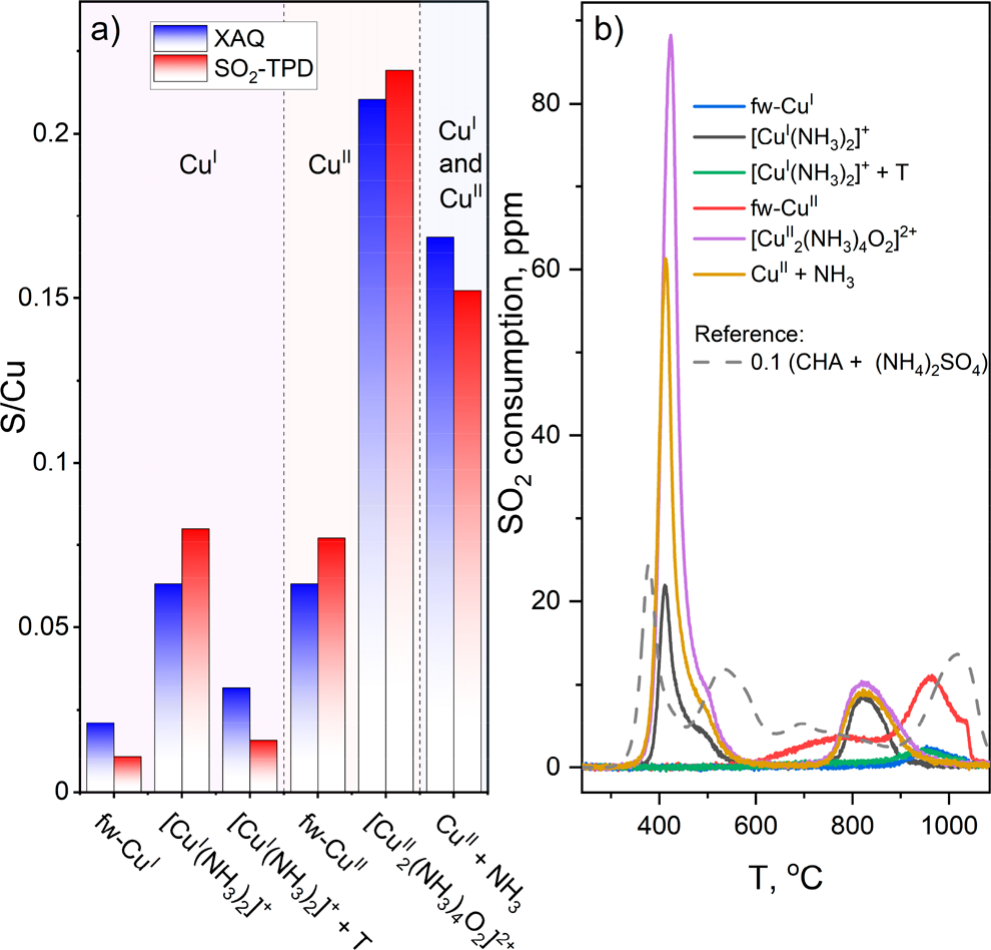
(a) S/Cu ratios in the samples after exposure to SO_2_ obtained from SO_2_-TPD and XAQ. (b) SO_2_-TPD profiles collected after exposure of the species obtained in procedures 1–6 to SO_2_ in comparison to a reference SO_2_-TPD curve of a CHA zeolite without Cu impregnated with 20 wt % (NH_4_)_2_SO_4_, downscaled ×10.

Interestingly, the sulfur content in the Cu^II^ + NH_3_ sample lies between those for samples with pure [Cu^I^(NH_3_)_2_]^+^ and [Cu^II^_2_(NH_3_)_4_O_2_]^2+^ species, which in combination with the linear combination fit shown in Figure 2 suggests that the reactivity of the Cu^II^(NH_3_)_*x*_O_*y*_ species obtained after Cu^II^ + NH_3_ treatment toward SO_2_ is similar to that of [Cu^II^_2_(NH_3_)_4_O_2_]^2+^.

By comparing the SO_2_-TPD curves of Cu-CHA samples with that of (NH_4_)_2_SO_4_, adsorbed on Cu-free CHA (Figure 3b), we can also deduce that the elevated sulfur content in the samples with the Cu^II^(NH_3_)_*x*_O_*y*_ species is due to the reactivity toward SO_2_ and not to the formation of (NH_4_)_2_SO_4_ in a reaction of SO_2_ with NH_3_ and NH_4_^+^ groups stored in the zeolite framework. For the adsorbed (NH_4_)_2_SO_4_, we observe SO_2_ desorption at around 380, 530, and 1000 °C (gray curve in Figure 3b). The desorption at 380 °C matches the known thermal decomposition of (NH_4_)_2_SO_4_;^32^ the other two peaks are probably due to the interaction of either (NH_4_)_2_SO_4_ or products of its decomposition with the zeolite, their precise interpretation being beyond the scope of the present argument. For all three Cu-CHA samples containing NH_3_ before exposure to SO_2_ ([Cu^I^(NH_3_)_2_]^+^, [Cu^II^_2_(NH_3_)_4_O_2_]^2+^, and (Cu^II^ + NH_3_) procedures), we observe SO_2_ desorption at around 420 °C (Figure 3b). As this does not match any of the observed desorption characteristics of (NH_4_)_2_SO_4_ in Cu-free Cu-CHA, the SO_2_-TPD feature at 420 °C reflects an interaction of Cu with SO_2_. Interestingly, the SO_2_-TPD curve for the sample with the dominant fw-Cu^II^ species shows a significant SO_2_ desorption peak close to 1000 °C, which, together with the lack of changes in Cu K-edge XANES upon exposure to SO_2_, indicates the formation of some sulfur deposits not directly coordinated to Cu.

The presence of Cu–N and Cu–O bonds in the [Cu^II^_2_(NH_3_)_4_O_2_]^2+^ complex has been independently confirmed by valence-to-core XES.^27,33,34^ XES spectra at different stages of pretreatment leading to the formation of [Cu^II^_2_(NH_3_)_4_O_2_]^2+^ dimers are reported in Figure 4. The origin of the Kβ′′ satellite peak is the transition from the ligand s orbitals to Cu 1s, which makes its position sensitive to the species directly coordinated to Cu and allows it to discriminate among Cu–O, Cu–N, and Cu–S bonds.^35−37^Figure 4 shows that after heating in O_2_ Cu is predominantly coordinated by oxygens (as expected for the fw-Cu^II^ species), whereas after exposure to NO + NH_3_ N ligands are dominating, as expected for a [Cu^I^(NH_3_)_2_]^+^ linear complex. After subsequent exposure to O_2_ and formation of [Cu^II^_2_(NH_3_)_4_O_2_]^2+^ dimers, the peak broadens, confirming the presence of both Cu–N and Cu–O bonds. These bonds remain after exposure to SO_2_, while no significant contribution from Cu–S bonds^38^ is observed, suggesting that the possible SO_2_ binding to the Cu is carried out through an oxygen atom.

**Figure 4:**
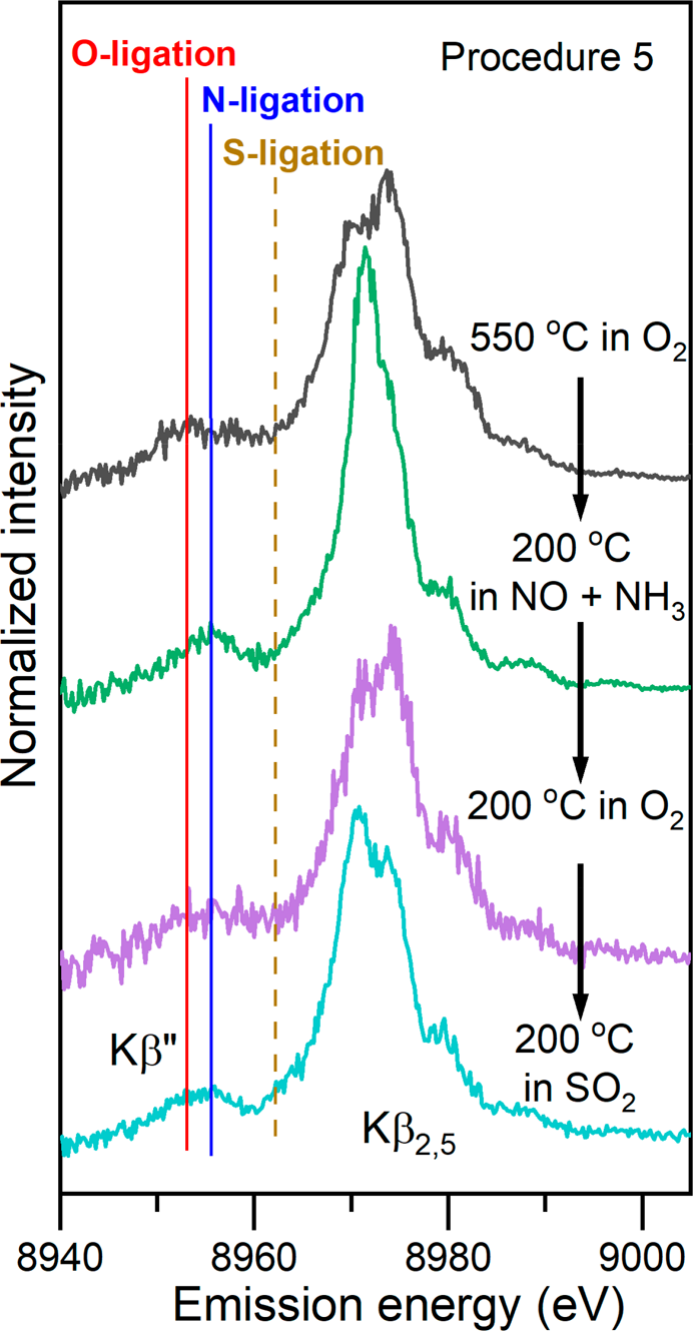
Background-subtracted Cu Kβ valence-to-core XES spectra for different stages of procedure 5 leading to the formation of the [Cu^II^_2_(NH_3_)_4_O_2_]^2+^ complex and its exposure to SO_2_.

In conclusion, the *in situ* XAS and XES measurements of different Cu intermediates formed in a Cu-CHA catalyst exposed to SO_2_ demonstrate that Cu^II^ species with mixed NH_3_ and O ligation of Cu are particularly reactive toward SO_2_, whereas Cu^I^ species and Cu^II^ without NH_3_ are much less affected by it. In particular, the [Cu^II^_2_(NH_3_)_4_O_2_]^2+^ complex, which is formed upon activation of O_2_ in the NH_3_-SCR cycle, shows a clear reaction with SO_2_, resulting in a partial reduction of the Cu^II^ and accumulation of sulfur in the zeolite. Therefore, we conclude that this reaction is responsible for the poisoning of Cu-CHA catalysts in NH_3_-SCR by SO_2_.

## Supporting Information Available

Experimental details, XANES linear combination fits before SO_2_ exposure, wavelet transform analysis of the sample heated in O_2_, and EXAFS fitting results (PDF)
