# Supplementary material for: SO2 Poisoning of Cu-CHA deNOx Catalyst: The Most Vulnerable Cu Species Identified by X-ray Absorption Spectroscopy
Source: JACS Au. 2022 Apr 11;2(4):787–92. doi: 10.1021/jacsau.2c00053 (PMC9088759; doi:10.1021/jacsau.2c00053)
Supplement: Supplementary file 1 — au2c00053_si_001.pdf [file au2c00053_si_001.pdf]

Supporting information

SO_2 poisoning of Cu-CHA deNO$_x$ catalyst: the most vulnerable Cu species identified by X-ray absorption spectroscopy

Anastasia Yu. Molokova[1,2], Elisa Borfecchia[2], Andrea Martini[2,3], Ilia A. Pankin[3], Cesare Atzori[1], Olivier Mathon[1], Silvia Bordiga[2], Fei Wen[4], Peter N.R. Vennestrøm[5], Gloria Berlier[2], Ton V.W. Janssens[5*] and Kirill A. Lomachenko[1*]

[1] European Synchrotron Radiation Facility, 71 avenue des Martyrs, CS 40220, 38043 Grenoble Cedex 9, France
[2] Department of Chemistry and NIS Centre, University of Turin, via Giuria 7,10125 Turin, Italy
[3] The Smart Materials Research Institute, Southern Federal University, Sladkova 174/28, 344090 Rostov-on-Don, Russia
[4] Umicore AG & Co, Rodenbacher Chaussee 4, 63457 Hanau, Germany
[5] Umicore Denmark ApS, Kogle Allé 1, 2970 Hørsholm, Denmark

Contents

1. Materials and methods 1
2. Experimental Procedures 2
3. XANES linear combination fit before SO_2 5
4. Wavelet transform analysis of the sample heated in O_2 7
5. EXAFS fitting 7

1. Materials and methods

The Cu-CHA catalyst was prepared by impregnation of the parent H-CHA zeolite material (Si/Al = 6.7) with the appropriate amount of an aqueous solution of Cu-nitrate. After impregnation, the samples were dried for 2 h at 90 °C, followed by 1 h of calcination at 500 °C in air to decompose the nitrates.

The XAS experiment was performed at BM23 beamline of the ESRF. The storage ring was operating in 4-bunch top-up mode at 20 mA maximum current. Measurements were performed in transmission mode at Cu K-edge, using a double-crystal Si(111) monochromator moving in a continuous mode. Acquisition time for one XAS spectrum was 2 min, energy range of 8800-10000 eV, energy step of 0.3 eV. A pair of flat Si mirrors at 2.8 mrad angle was used for harmonic rejection. Ionization chambers were used to detect incident and transmitted photons. I_0 chamber was filled with 1.57 bar N_2, I_1 and I_2 with 0.33 bar Ar, all three of them topped-up with He to the total pressure of 2 bar(a). The spectra of the Cu reference foil were measured together with the sample and then used for alignment. The XAS data were analysed using Athena program from Demeter package[1] and Larch[2] code.

The X-ray adsorbate quantification (XAQ) data, reported in Figure 3 of the main text, were obtained by monitoring the change of the total absorption coefficient before Cu K edge (at 8885 eV, averaging the region of 8820-8950 eV) during the interaction SO_2. The change of the absorption coefficient was attributed to the adsorption of SO_2. The corresponding number of moles of SO_2 per unit area of the pellet was calculated by XAFS

mass code[3] and, knowing the parameters of the sample pellet (mass, surface density and Cu content), converted into Cu/S ratio.

Cu Kβ valence to core (vtc) XES measurements were carried out at ID26 beamline of the ESRF. The storage ring was operating in uniform top-up mode at 200 mA maximum current. The measurements were done using the five analyser crystal spectrometer in vertical Rowland geometry. Incident radiation energy was fixed at 9050 eV, set by a flat Si (311) double-crystal monochromator. Scans of emitted radiation energy were performed using the (800) reflection of five identical Ge (100) analyser crystals. The crystals were spherically bent following the Johann scheme to focus the fluorescence radiation on the APD detector. Sample-analyzer distance was ca. 1m. The overall energy resolution of ca. 1.7 eV estimated by the FWHM of the elastic peak. Between the cell, analyzer crystals and detector a He-filled bag was placed in order to minimize X-ray absorption along the sample-analysers-APD path. In order to decrease radiation damage, the incident beam was attenuated by a factor of 10. Vtc XES data were collected integrating 0.5 s per point, each point collected at the fresh spot of the sample. After each vtc-XES scan a correction scan was performed with the same integration time, measuring intensity of Kβ1,3 main line in the same spots on the sample, which was then used for the normalization of vtc XES data. A background function was subtracted from the XES spectra. The latter was modelled in the following way: the region before and after XES Kβ vtc line was fitted with a sum of three Pseudo Voigt functions to reproduce the downward trend of the Kβ main maximum.

For both XAS and XES experiments, the samples of Cu-CHA catalyst were prepared in a form of self-supported pellets of 13 mm diameter. The pellet mass of 104 mg was optimised by XAFSmass code[3] to maximise the signal-to-noise ratio in transmission XAS measurements. Resulting Cu K-edge edge jump was ca. 0.6. In both experiments the measurements were conducted in flow conditions (total flow in SO_2 was 50 ml/min, total flow in other gas mixtures was 100 ml/min) using Microtomo reactor cell[4] of the ESRF and mobile gas mixing setup for both experiments. The outlet gas composition was monitored using a mass spectrometer (Balzers Prisma).

The SO_2-TPD was performed with the samples of the same material sulfated following the same procedures as employed in the XAS experiment. The SO_2-TPD was carried out with simultaneous thermal analyzer (STA 449 F3 Jupiter, NETZSCH), which is coupled with an infrared (FTIR) spectrometer (MKS 2030, MKS Instruments) for evolved gas analysis. The sample was first kept in nitrogen gas flow 100 ml/min at 50°C for 30 mins and then heated up to 1200°C with 10 K/min. The sample mass was 25 mg.

2. Experimental Procedures

The goal of each experimental procedure was to induce a preferential formation of one of Cu intermediates in the SCR reaction. To obtain them each sample was activated in oxygen and exposed to the suitable conditions. The detailed schemes containing the information on the temperatures, gas compositions and timing are presented in Figures S1-S6.

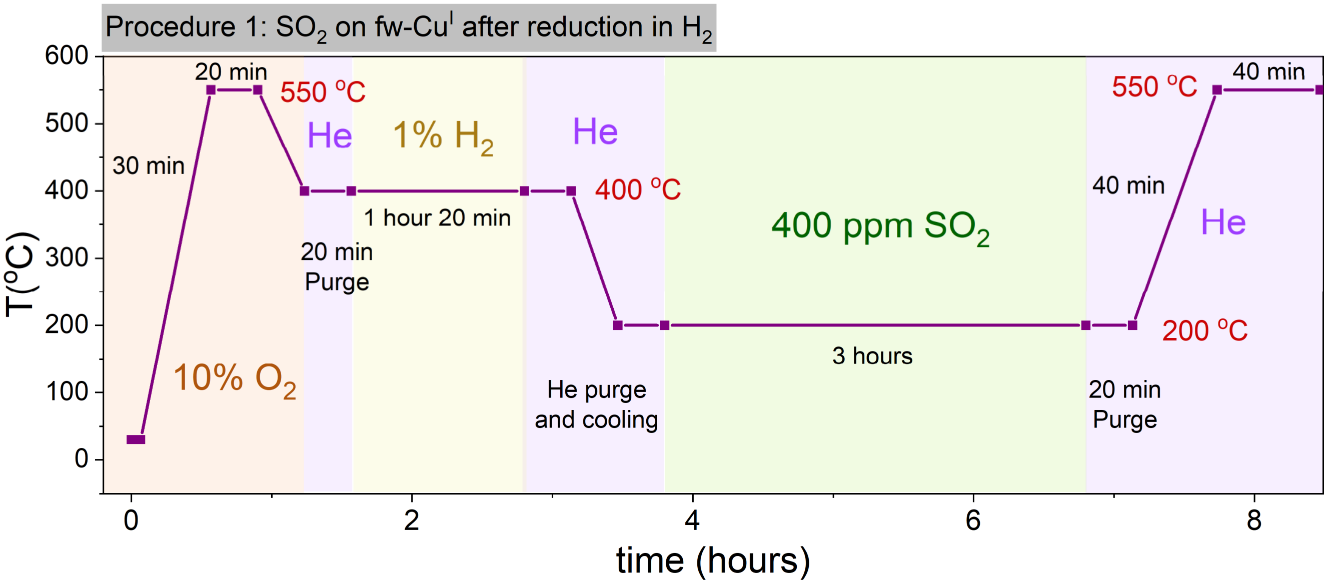

Figure S1 Scheme of the followed experimental protocol comprising the pre-treatment Procedure 1 leading to the formation of fw-Cu^{I} species prior to exposure to SO_2

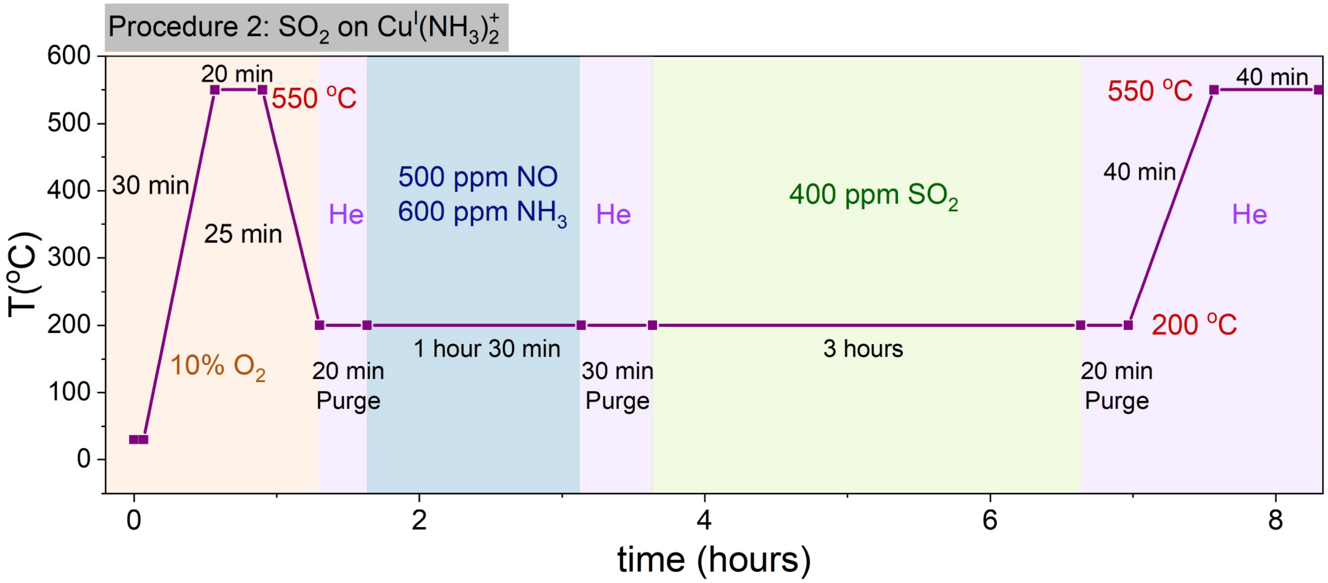

Figure S2 Scheme of the experimental protocol comprising the pre-treatment Procedure 2, leading to the formation of mobile $[Cu^{I}(NH_3)_2]^+$ complexes prior to exposure to SO_2

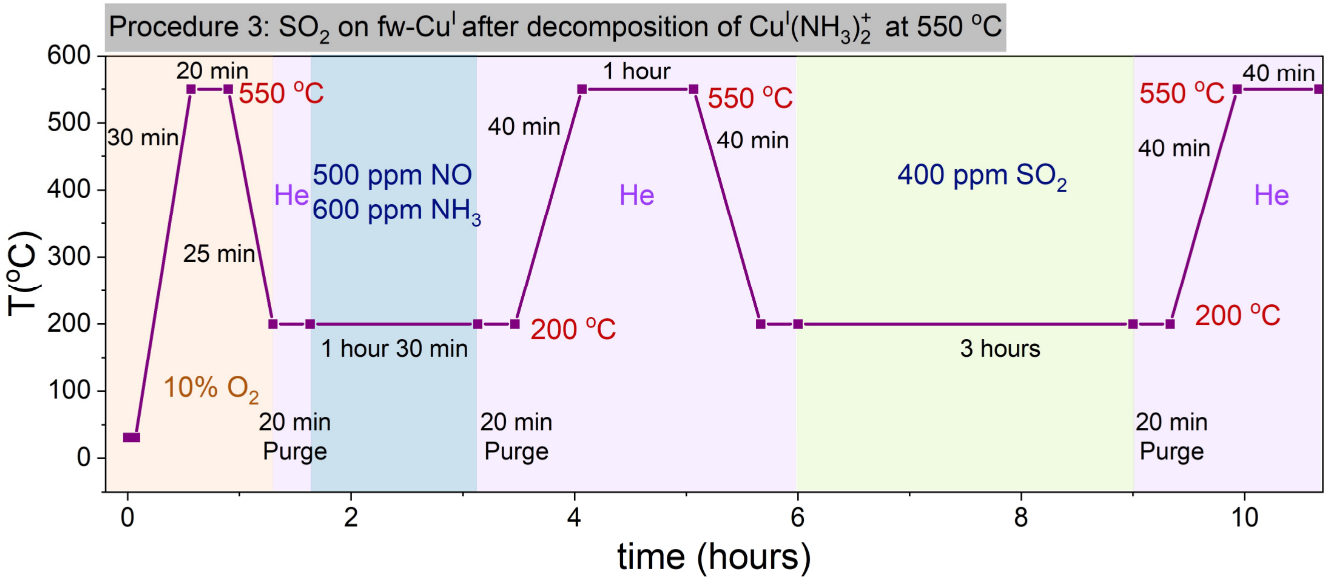

Figure S3 Scheme of the experimental protocol comprising the pre-treatment Procedure 3 leading to the formation of fw-Cu^{I} species prior to exposure to SO_2

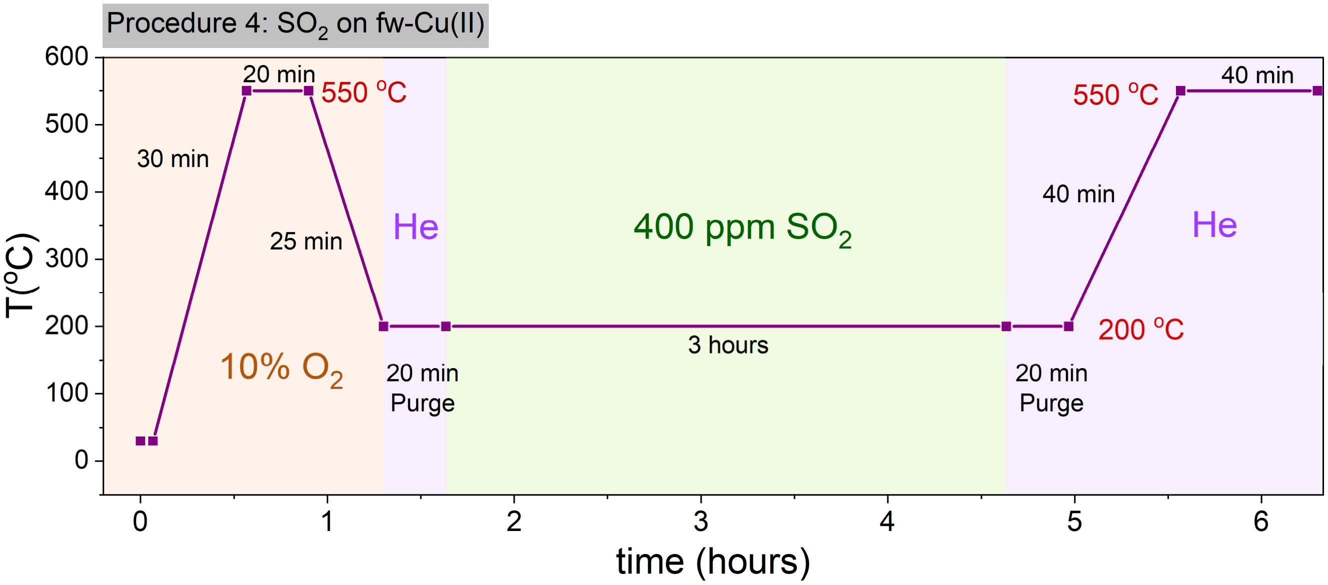

Figure S4 Scheme of the experimental protocol comprising the pre-treatment Procedure 4 leading to the formation of fw-Cu^{II} species prior to exposure to SO_2

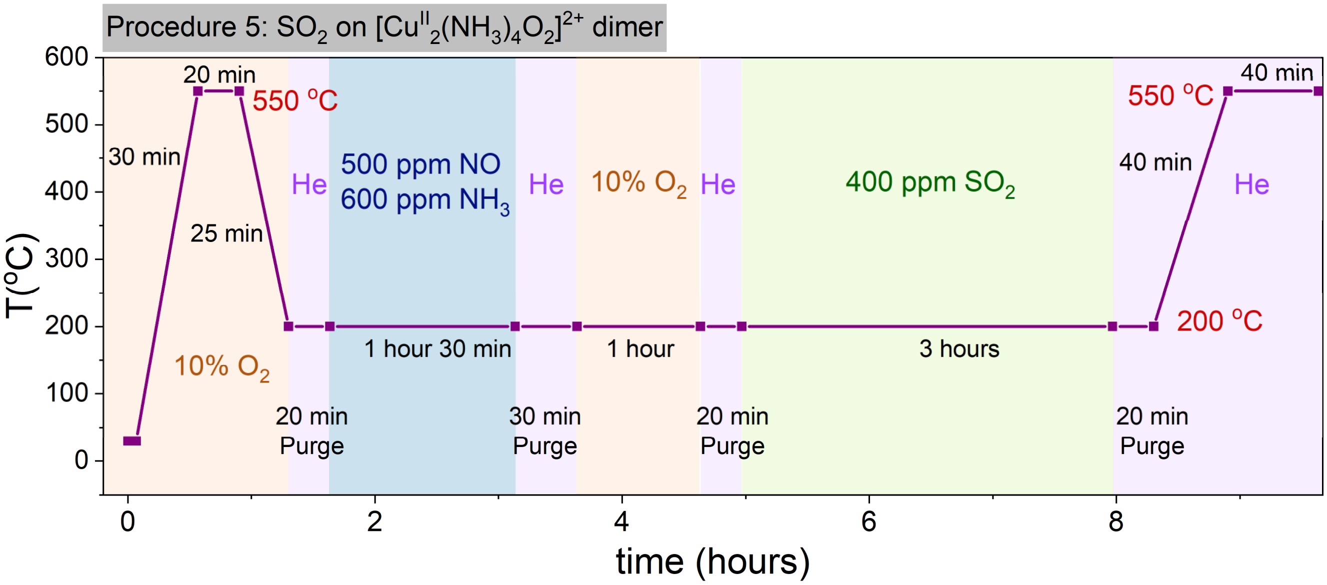

Figure S5 Scheme of the experimental protocol comprising the pre-treatment Procedure 5 leading to the formation of mobile $[Cu^{II}_2(NH_3)_4O_2]^{2+}$ dimers prior to exposure to SO_2

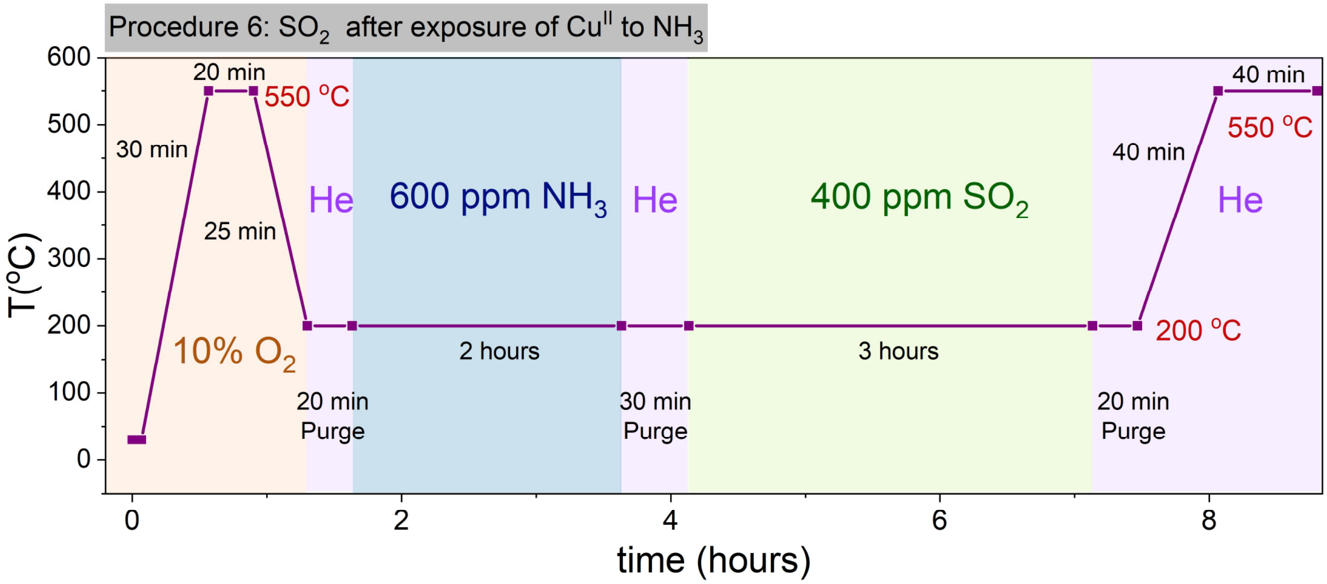

Figure S6 Scheme of the experimental protocol comprising the pre-treatment Procedure 6 leading to the formation of mixture of NH_3-coordinated Cu^I/Cu^{II} species prior to exposure to SO_2

3. XANES linear combination fit before SO_2

In this section we present the comparison between different linear combination fits (LCF) for the Cu species obtained in Procedure 6 (Cu^{II} + NH_3). In Figure S7 we compare the linear combination fits results with different reference components. One component (linear diamine complex $[Cu^I(NH_3)_2]^+$) is the same for all the fits. As the

second component, we use the spectrum of oxygen-bridged diamine dicopper complex $[Cu^{II}_2(NH_3)_4O_2]^{2+}$ (Figure S7a), the spectrum of tetraamine complex in solution $[Cu^{II}(NH_3)_4]^{2+}$ (Figure S7b) and the spectrum of tetraamine copper sulfate monohydrate $[Cu^{II}(NH_3)_4]SO_4 \cdot H_2O$ (Figure S7c). The best agreement with the data was obtained for the fit with oxygen-bridged diamine dicopper complex $[Cu^{II}_2(NH_3)_4O_2]^{2+}$ as the second reference.

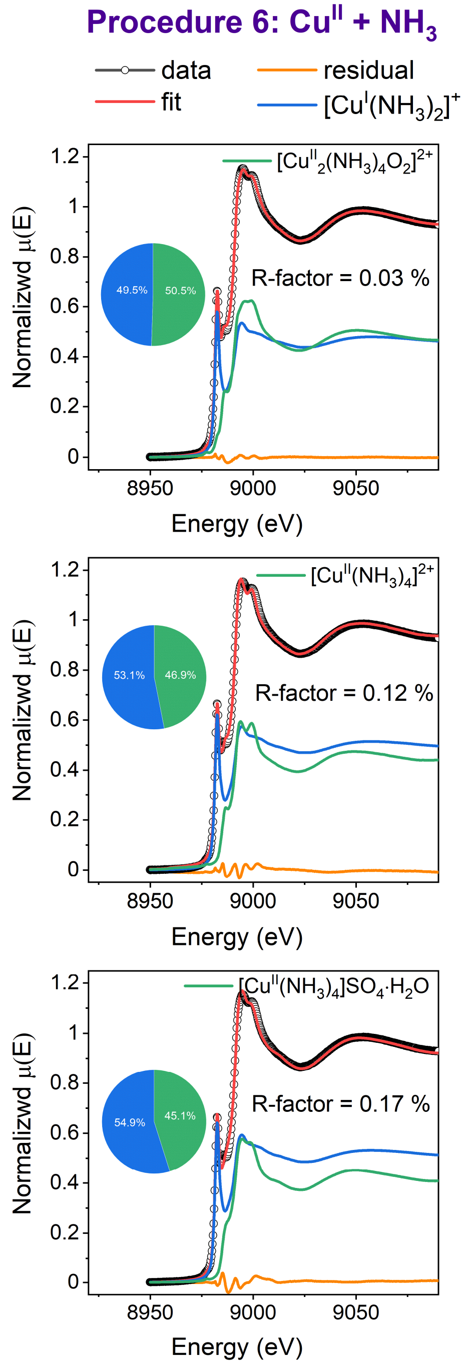

Figure S7. Comparison between LCF results with different references for the XANES spectrum collected after pre-treatment in Procedure 6; the reference spectra are the spectrum of linear diamine complex $[Cu^{I}(NH_3)_2]^{+}$ (the same for all fits) and (a) oxygen-bridged diamine dicopper complex $[Cu^{II}_2(NH_3)_4O_2]^{2+}$, (b) tetraamine copper complex in solution $[Cu^{II}(NH_3)_4]^{2+}$, (c) tetraamine copper sulfate monohydrate $[Cu^{II}(NH_3)_4]SO_4 \cdot H_2O$.

4. Wavelet transform analysis of the sample heated in O_2

Wavelet transform (WT) analysis is applied to EXAFS data for distinguishing the contributions of light and heavy atoms in the EXAFS signal [5, 6]. The distinction is coming from the dependence of backscattering amplitude factors on the atomic number Z. As a result, the contributions of heavier atoms are localized at higher k. With WT it is possible to represent the signal in both R- and k-space making possible to assign it to the scattering paths with a better precision.

Following the procedure analogous showed in works [6, 7] we used WT for identifying the presence of Cu in Cu-CHA after initial heating in O_2 to 550 °C and subsequent cooling to 200 °C. The Cu-Cu contribution is expected to be localized at ca. 6-7 Å^{-1}, according to the calculations of the backscattering amplitude factors[2]. Such contribution is indeed visible in WT map of the examined Cu-CHA sample (Figure S8). Together with Z-Cu^{II}(OH) species, the dimers, that are usually oxygen-bridged, may constitute a necessary stock of oxygen needed for the formation of mixed-ligand $Cu^{II}(NH_3)_xO_y$ complexes after the subsequent treatment in NH_3 at 200 °C in Procedure 6.

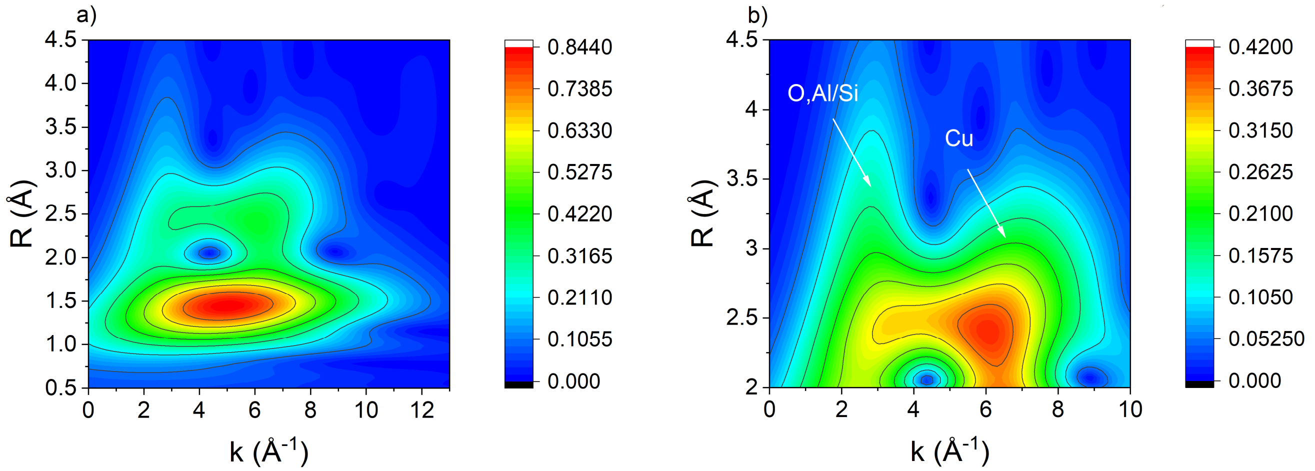

Figure S8. Full-range WT representation of the EXAFS signal for the Cu-CHA sample measured at 200 ºC after heating to 550 ºC in 10% O_2/He and cooling to 200 ºC; b) Magnification of the high-R WT region in ranges Δk (0-10) Å^{-1} and ΔR (2-4.5) Å. WT sub-lobes stemming from O, Al/Si and Cu atomic neighbours are indicated by white arrows and labels.

5. EXAFS fitting

The composition of the Cu species proposed in the LCF section was supported by EXAFS fitting results (Table S1). The fitting was performed using the Athena and Artemis software from the Demeter [1] package. For the complex $[Cu^{II}_2(NH_3)_4O_2]^{2+}$ from Procedure 5 before SO_2 the fitting model was chosen according to the structure

proposed in literature[8]. Although it was shown[9] that this state can contain a small fraction (around 10%) of Cu^I, here we assumed a "pure Cu^{II}" approximation. For the mixed species after (Cu^{II} + NH_3) treatment in Procedure 6 before SO_2 the coordination numbers from theoretical models were multiplied by the coefficients obtained from the linear combination fitting described above. For all fits, coordination numbers were fixed. Path lengths and Debye-Waller factors were fixed for the paths taken from previously done fits of the corresponding components, other path lengths and DW factors were fitted. Figure S9 shows the resulted fits in k- and R-space.

Table S1. The results of EXAFS fitting. Fitting ranges: k-range = 3-13 $Å^{-1}$, R-range = 1.15-3 Å. Colour codes of the paths correspond to the following species: yellow: $[Cu^{II}_2(NH_3)_4O_2]^{2+}$, light-blue: $[Cu^I(NH_3)_2]^+$. Parameters without errors in parentheses were taken from the fit of the corresponding component and fixed in the fit here

Cu-species	Path	Parameters	R-factor, %
$[Cu^{II}_2(NH_3)_4O_2]^{2+}$	Cu-O	N = 2, σ^2 = 0.008(1) $Å^2$, R = 1.921(1) Å	0.2
(Procedure 5)	Cu-N	N = 2, σ^2 = 0.006(1) $Å^2$, R = 2.016(1) Å	
(Cu^{II} + NH_3)	Cu-N	N = 2 · 0.495*, σ^2 = 0.008 $Å^2$, R = 1.91 Å	0.8
(Procedure 6)	Cu-N	N = 2 · 0.505*, σ^2 = 0.003(2) $Å^2$, R = 2.03(2) Å	
	Cu-O	N = 2 · 0.505*, σ^2 = 0.004(2) $Å^2$, R = 1.92(2) Å	

* coefficients for N are taken from XANES LCF results (Figure 2 Main text)

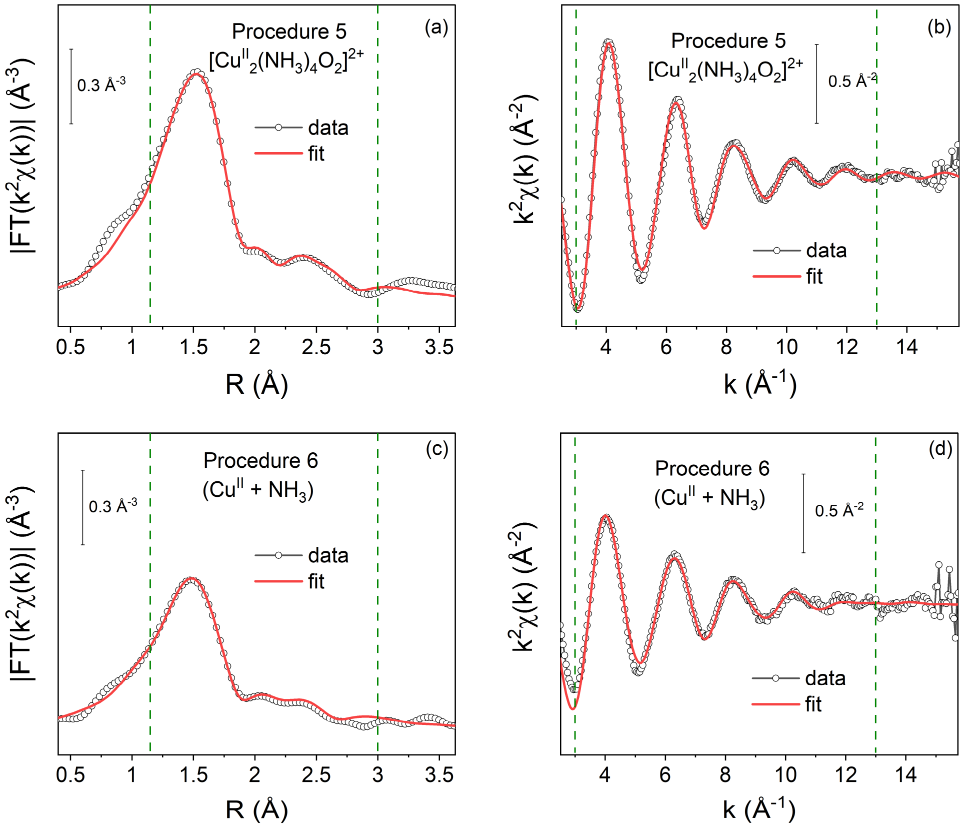

Figure S9. EXAFS fitting results for $[Cu^{II}_2(NH_3)_4O_2]^{2+}$ complex (a, b) and the outcome of (Cu^{II}+NH_3) treatment (Procedure 6) (c, d) after pre-treatment procedures in R- (left panels) and k-space (right panels). Green dashed lines show the fitting ranges.

References

(1) Ravel, B.; Newville, M., ATHENA and ARTEMIS Interactive Graphical Data Analysisusing IFEFFIT. *Phys. Scr.* **2005,** *T115*, 1007–1010.

(2) Newville, M. In *Larch: An Analysis Package for XAFS and Related Spectroscopies*, 15th International Conference on X-Ray Absorption Fine Structure (XAFS), Beijing, PEOPLES R CHINA, Jul 22-28; Beijing, PEOPLES R CHINA, 2012.

(3) Klementiev, K.; Chernikov, R., XAFSmass: a program for calculating the optimal mass of XAFS samples. *J. Phys. Conf. Ser.* **2016,** *712*, 012008.

(4) Bellet, D.; Gorges, B.; Dallery, A.; Bernard, P.; Pereiro, E.; Baruchel, J., A 1300 K furnace for in situ X-ray microtomography. *J. Appl. Crystallogr.* **2003,** *36*, 366-367.

(5) Timoshenko, J.; Kuzmin, A., Wavelet data analysis of EXAFS spectra. *Comput. Phys. Commun.* **2009,** *180* (6), 920-925.

(6) Martini, A.; Signorile, M.; Negri, C.; Kvande, K.; Lomachenko, K. A.; Svelle, S.; Beato, P.; Berlier, G.; Borfecchia, E.; Bordiga, S., EXAFS wavelet transform analysis of Cu-MOR zeolites for the direct methane to methanol conversion. *Phys. Chem. Chem. Phys.* **2020,** *22* (34), 18950-18963.

(7) Martini, A.; Bugaev, A. L.; Guda, S. A.; Guda, A. A.; Priola, E.; Borfecchia, E.; Smolders, S.; Janssens, K.; De Vos, D.; Soldatov, A. V., Revisiting the Extended X-ray Absorption Fine Structure Fitting Procedure through a Machine Learning-Based Approach. *J. Phys. Chem. A* **2021,** *125* (32), 7080-7091.

(8) Negri, C.; Selleri, T.; Borfecchia, E.; Martini, A.; Lomachenko, K. A.; Janssens, T. V. W.; Cutini, M.; Bordiga, S.; Berlier, G., Structure and Reactivity of Oxygen-Bridged Diamino Dicopper(II) Complexes in Cu-Ion-Exchanged Chabazite Catalyst for NH3-Mediated Selective Catalytic Reduction. *J. Am. Chem. Soc.* **2020,** *142* (37), 15884-15896.

(9) Negri, C.; Martini, A.; Deplano, G.; Lomachenko, K. A.; Janssens, T. V. W.; Borfecchia, E.; Berlier, G.; Bordiga, S., Investigating the role of Cu-oxo species in Cu-nitrate formation over Cu-CHA catalysts. *Phys. Chem. Chem. Phys.* **2021,** *23* (34), 18322-18337.
